# A Flagella Hook Coding Gene *flgE* Positively Affects Biofilm Formation and Cereulide Production in Emetic *Bacillus cereus*

**DOI:** 10.3389/fmicb.2022.897836

**Published:** 2022-06-10

**Authors:** Yangfu Li, Nuo Chen, Qingping Wu, Xinmin Liang, Xiaoming Yuan, Zhenjun Zhu, Yin Zheng, Shubo Yu, Moutong Chen, Jumei Zhang, Juan Wang, Yu Ding

**Affiliations:** ^1^Department of Food Science and Technology, Institute of Food Safety and Nutrition, Jinan University, Guangzhou, China; ^2^State Key Laboratory of Applied Microbiology Southern China, Key Laboratory of Agricultural Microbiomics and Precision Application, Ministry of Agriculture and Rural Affairs, Guangdong Provincial Key Laboratory of Microbial Safety and Health, Institute of Microbiology, Guangdong Academy of Sciences, Guangzhou, China; ^3^College of Food Science, South China Agricultural University, Guangzhou, China

**Keywords:** *Bacillus cereus*, biofilm, flagella, cereulide, motility

## Abstract

*Bacillus cereus*, an important foodborne pathogen, poses a risk to food safety and quality. Robust biofilm formation ability is one of the key properties that is responsible for the food contamination and food poisoning caused by *B. cereus*, especially the emetic strains. To investigate the mechanism of biofilm formation in emetic *B*. *cereus* strains, we screened for the mutants that fail to form biofilms by using random mutagenesis toward *B. cereus* 892-1, an emetic strain with strong biofilm formation ability. When knocking out *flgE*, a flagellar hook encoding gene, the mutant showed disappearance of flagellar structure and swimming ability. Further analysis revealed that both pellicle and ring presented defects in the null mutant compared with the wild-type and complementary strains. Compared with the flagellar paralytic strains **Δ** *motA* and **Δ** *motB*, the inhibition of biofilm formation by **Δ** *flgE* is not only caused by the inhibition of motility. Interestingly, **Δ** *flgE* also decreased the synthesis of cereulide. To our knowledge, this is the first report showing that a flagellar component can both affect the biofilm formation and cereulide production in emetic *B. cereus*, which can be used as the target to control the biohazard of emetic *B. cereus*.

## Introduction

*Bacillus cereus*, a Gram-positive, spore-forming, and facultative anaerobe with flagella, is an important pathogen associated with foodborne outbreaks worldwide and causing clinical manifestations like gastroenteritis, emesis, fulminant bacteremia, bone infection, and brain abscess (Majed et al., 2016; Enosi Tuipulotu et al., 2021). Two types of food poisoning can be caused by *B. cereu*s, including diarrhea and vomiting (Zhou et al., 2019), with the latter one triggered by cereulide, which is preformed in food (Naranjo et al., 2011). Although symptoms caused by cereulide are usually self-limiting (Rouzeau-Szynalski et al., 2020), fatal cases have been reported (Mahler et al., 1997; Dierick et al., 2005; Shiota et al., 2010; Naranjo et al., 2011).

Cereulide is synthesized by enzymes encoded by the *ces* gene cluster located on a 270-kb mega-plasmid, named pCER270, which displayed a high similarity in sequence with the plasmid pXO1 in *Bacillus anthraci*s (Rasko et al., 2007). After ingestion, cereulide is absorbed in the intestine and distributed throughout the body, which can be detected in the stomach, spleen, liver, kidney, muscles, and fat tissues, or even crossed the blood-brain barrier (Bauer et al., 2018). Chronic cereulide exposure induced endoplasmic reticulum stress response, intestinal inflammation, dysregulation of intestinal flora, and inhibition of serotonin biosynthesis (Lin et al., 2021). Notably, cereulide is stable to trypsin, acid, and heat (121°C for 2 h) (Dommel et al., 2010; Jovanovic et al., 2021), so conventional food processing conditions are unable to inactivate it. Emetic strains that produce cereulide are ubiquitous in different kinds of food, of which dairy products account for a relatively high proportion (Shaheen et al., 2006; Messelhäusser et al., 2010; Owusu-Kwarteng et al., 2017; Gao et al., 2018), e.g., emetic strains are found in 21% raw milk samples, in which 1,140 ng/mL cereulide can be detected (Rajkovic et al., 2006; Owusu-Kwarteng et al., 2017). It was reported cereulide caused food poisoning to an adult and rapid death of a healthy 1-year-old boy (Shiota et al., 2010) at a very low concentration (4 ng/mL in the serum). Since food poisoning outbreaks caused by emetic *B. cereus* resulted in severe cases, the presence of emetic *B. cereus* in the food and food processing chain is of great concern to food safety. Therefore, it is very important to eliminate emetic *B. cereus* in food.

Biofilm is a sessile community of microbes that adhere to the surface of abiotic or living tissue and are coated with the extracellular polymer matrix (EPS) produced by the microbes to adapt to the living environment (Costerton et al., 1999). Because of the shelter of EPS, bacteria can survive under stress conditions, including disinfectants and antimicrobials (biocides) in the biofilm lifestyle than in the planktonic form (Alvarez-Ordóñez et al., 2019). Biofilm is also the reservoir of spores, which are more resistant to heat, acid, and low water activity, and the biofilm lifestyle provides a higher proportion of spores than in planktonic culture (Ribeiro et al., 2019; Rouzeau-Szynalski et al., 2020). Since the clean-in-place (CIP) system commonly used in the food processing chain cannot eliminate spores (Thomas and Sathian, 2014), *Bacillus* becomes the dominant taxa in the milk processing chain (Kable et al., 2019). Once the biofilm is formed, it is inevitable to cause contamination in the processing environments and final products (Ostrov et al., 2016; Silva et al., 2018).

*Bacillus subtilis* is a model organism for studying regulatory networks directing biofilm formation among Gram-positive and spore-forming bacteria. Genes and regulatory pathways controlling biofilm formation have been well studied in *B. subtilis* (Vlamakis et al., 2013; Mielich-Süss and Lopez, 2015). In contrast to *B. subtilis*, few genes were involved in biofilm formation have been characterized in *B. cereus* and the regulatory mechanisms that control biofilm formation are poorly understood. *Bacillus cereus* produces different forms of biofilms including submerged biofilm, pellicle, and ring, that differ in their architecture and may be regulated by different genetic determinants (Wijman et al., 2007; Caro-Astorga et al., 2014; Gao et al., 2015). Previous studies showed that Spo0A and CodY act as key regulators in biofilm formation in *B. cereus* (Lindbäck et al., 2012; Gao et al., 2015). Besides, motility and flagella may involve in biofilm formation in *B. cereus* (Houry et al., 2010). Although a variety of genes were found by a genome-wide investigation with random mutagenesis and RNA sequencing, current knowledge about *B. cereus* biofilm formation, especially in the emetic strains, is still largely unknown (Yan et al., 2017).

In this study, we constructed a transposon mutagenesis library of an emetic *B. cereus* strain 892-1 with strong biofilm-forming ability, which was isolated from pasteurized milk (Gao et al., 2018). By high-throughput screening of biofilm-defective mutants, we successfully identified a mutant named 3-86, which showed a significant defect in biofilm formation. Further analysis found that the insertion site of the transposon is a flagellar hook encoding gene *flgE*, which not only has a positive regulation function in biofilm formation but also affects cereulide production. To our knowledge, this is the first time to illustrate the function of a flagellar hook encoding gene *flgE* on both biofilm formation and cereulide production in emetic *B. cereus*. Therefore, this study may provide a new strategy for the control of food contamination and poisoning incidents caused by emetic *B. cereus*.

## Materials and Methods

### Bacterial Strains and Culture Condition

*Bacillus cereus* 892-1 and its derivatives were cultured in tryptic soy broth (TSB; Guangdong Huankai Co., Ltd., Guangzhou, China) at 37°C, 200 rpm, or on nutrient agar plates (Guangdong Huankai Co., Ltd.) at 37°C. *Escherichia coli* strains were grown at 37°C in luria-bertani broth (LB; Guangdong Huankai Co., Ltd.). When needed, antibiotics were added at the following concentrations: 5 μg/mL of erythromycin, 17 μg/mL chloramphenicol for the growth of *B. cereus*, and 100 μg/mL of ampicillin for the growth of *E. coli*. A list of strains and plasmids used in this work is provided in Supplementary Table 1. Oligonucleotides are listed in Supplementary Table 2.

### Construction of Transposon Mutagenesis Library

**FIGURE 1:**
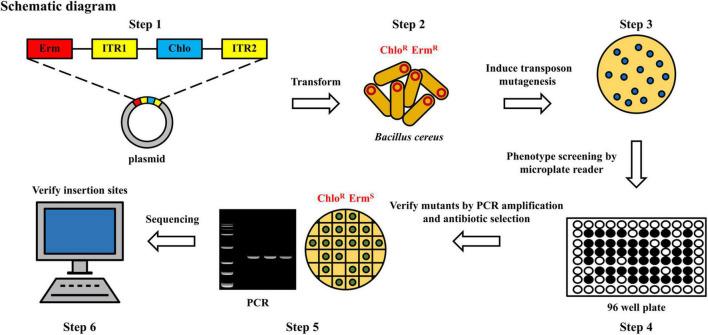
Flow chart of construction of random mutagenesis library to screen the potential mutants with defects in biofilm formation. Erm, erythromycin; Chlo, chloromycin; R, resistant; S, sensitive; ITR1/ITR2, transposon.

The construction and screening steps of a transposon mutagenesis library were depicted in Figure 1. The plasmid pMarA (Gao et al., 2019) carrying a mariner-based transposon Tn*YLB*-1 was used as the backbone. To replace the selectable marker kanamycin resistance cassette, the chloromycin resistance cassette was amplified from pBAD33 (Guzman et al., 1995). The newly generated plasmid, named pMarA-cat, was transformed into the strain 892-1 by electroporation, followed by the selection for both Erm^R^ (erythromycin-resistant) and Chlo^R^ (chloramphenicol-resistant) colonies at 28°C. Positive transformants were inoculated at 37°C, 200 rpm overnight to induce transposon-mediated mutagenesis. Fifty microliters of diluted cultures (1:100,000, v:v) were then spread onto LB agar plates containing chloramphenicol and incubated at 48°C for 10 h to induce plasmid suicide. The biofilm phenotypes of each mutant was screened by a microplate reader (Gen5™, BioTek, Winooski, VT, United States). Then, potential mutants with altered phenotypes were verified by antibiotic selection, which are resistant to chloramphenicol (Chlo^R^) and sensitive to erythromycin (Erm*^S^*). To select mutants containing a transposon insertion, a quick DNA extraction method was used. Briefly, a single colony was suspended into 30 μL ddH_2_O in a 1.5 mL tube and then ultrasonically treated at 40 kHz at 25°C for 5 min. Then, the mixture was centrifuged at 10,000 *g*, 25°C for 1 min. Afterward, the upper aqueous phase was carefully transferred to a new 1.5 mL tube without disturbing the pellet. The DNA samples was tested by the polymerase chain reaction (PCR). To confirm the transposon insertion site, restriction endonuclease *Taq* I (ER0671, Thermo Fisher Scientific Inc., Waltham, MA, United States) was used to cut the genomic DNA. The genome fragments were self-ligated by T4 DNA ligase at 25°C for 10 min (EL0014, Thermo Fisher Scientific, Waltham, MA, United States), and reverse PCR was performed using primer OIPCR-1/2 to amplify the sequence inserted by Tn*YLB*-1 transposon. After sequencing, the insertion sites were identified by local blast (ncbi-blast-2.12.0+ -win64).

### Construction of Deletion Mutant and Complementary Strain

Different strains was constructed as described previously (Qi et al., 2011). Plasmid pHT304-TS (Zhu et al., 2011) was used to construct mutants by homologous recombination. Recombinant plasmids were generated by in-fusion cloning (Ma et al., 2019). Briefly, the vector was linearized by restriction endonuclease *EcoR* I and *Sal* I (1611 and 1636, Takara, Shiga, Japan) digestion. The upstream and downstream fragments were amplified by using genome DNA as the template. 5′ end of the forward and reverse primers of inserts were amplified by PCR with 15-20 bp homolog fragments of linearized vector. Then, the mixture of inserts, linearized vector, and 2 × Hieff Clone^®^ Enzyme Premix (10912 and 10911, Yeasen, Shanghai, China) was incubated at 50°C for 1 h by using a thermo-cycler (C1000 Touch, Bio-Rad, Hercules, CA, United States). The mixture was then transformed into *E. coli* DH5α by thermal shock directly.

For the construction of mutants, including Δ*flgE*, Δ*motA*, and Δ*motB*, recombinant plasmids were transformed into *B. cereus* 892-1 by electroporation as mentioned above. One milliliter SOC media was immediately added to suspend the bacteria and the mixture was then incubated at 30°C, 200 rpm for 3 h. After incubation, the bacteria were collected by centrifugation at 25°C 5,000 *g* for 2 min and then spread on an LB agar plate with erythromycin. Plates were incubated overnight in an incubator at 30°C and potential transformants were confirmed by PCR. For gene knockout, positive transformants were transferred to media with erythromycin and incubated at 42°C, 200 rpm for 6–12 h, and this process was repeated 6 times. Then, strains were incubated at 30°C, 200 rpm for 6–12 h for 9–12 times and spread on LB agar plates without antibiotics. Colonies were then transferred on LB agar plates with antibiotics, and the ones that could not grow on the plates were detected by PCR and sent for sequencing. For the construction of complementary strains, plasmid pHT304 (Arantes and Lereclus, 1991) was used. The gene was cloned into plasmid pHT304 by in-fusion cloning as described above. Recombinant plasmids and electroporation were followed as described above and positive transformants were used for the following experiments.

### Growth Curve of Different Strains

To evaluate the effect of gene deletion and complementation on bacterial growth, overnight cultures were diluted 1,000-fold (v:v) into fresh TSB broth, and then 200 μL of bacterial suspension was added to the wells of a 96-well plate. In total, three biological repeats with six technical repeats each were performed. Bacterial growth was monitored by measuring the optical density at OD_600_ of each well at 37°C every 30 min for 12 h by using a microplate spectrophotometer (EPOCH2, Biotek, Vermont, United States) and the data were analyzed by GraphPad Prism (v8.0.2) to generate XY plots.

### Biofilm Formation Assay

For pellicle formation analysis, *B. cereus* strains were grown overnight at 37°C, 200 rpm. Five microliters of overnight culture were inoculated into 5 mL TSB medium in a test tube, which was then statically incubated at 37°C for 12 h. The formation of the pellicle was recorded by a Nikon D750 camera. Evaluation of the ring part was performed as previously described with minor modifications (Stepanović et al., 2000). Briefly, Bacteria (3.3 × 10^5^ cfu/mL) were inoculated into 200 μL fresh TSB medium in 96-well polystyrene plates (Costar, Washington, DC, United States), and incubated at 37°C for 12 h statically. Then, planktonic cells were poured out, and plates were washed three times with ddH_2_O. The remaining attached biofilms were dried and fixed with 210 μL of 95% methanol per well for 15 min. After drying, 210 μL of crystal violet (0.1%; w/v) was added to each well and incubated for 15 min to stain biofilms attached to the well surface. After briefly washing three times and drying, the crystal violet was dissolved in 220 μL of 30% acetic acid, and staining levels were assessed by measuring absorbance at 590 nm (A_590_).

### Scanning Electron Microscopy

Overnight cultures were diluted to an OD_600_ of 0.001 in TSB broth and biofilms were grown on 8 mm × 8 mm glass coverslips (WHB-48-CS, WHB, Shanghai, China) in 12-well plates (Costar, Washington, DC, United States) for 12 h at 37°C. Biofilms formed on the surface of the cell slide were fixed with 3% (w/v) glutaraldehyde overnight at 4°C. Samples were then dehydrated with a graded ethanol series, dried, sputter-coated with gold, and imaged by a scanning electron microscope (Hitachi S-3000N, Tokyo, Japan) operating at 20 kV and 83 μA.

### Confocal Laser Scanning Microscopy Analysis

The biofilm structure was visualized by confocal laser scanning microscopy (CLSM) as described previously (Zhao et al., 2022) with minor modifications. Overnight cultures were diluted 1,000 (v:v) times in 50 mL fresh TSB broth and biofilms were grown in a beaker (100 mL volume), which were then observed using a confocal laser scanning microscope (ZEISS LSM700, Oberkochen, Germany). Biofilms without planktonic cells were stained using SYTO^®^ 9 (Thermo Fisher Scientific Inc., Waltham, MA, United States) at 25°C in the dark for 2 min. Biofilms were then visualized using a CLSM by a 20× objective lens with excitation at 488 nm and emission at 500–550 nm. The images were processed by using the Zeiss ZEN (v3.5).

### Transmission Electron Microscopy

Different samples were examined by transmission electron microscopy (TEM; Tecnai G2 F20 S-TWIN, Thermo Fisher Scientific Inc., Waltham, MA, United States) for the appearance of flagella. Overnight cultures were diluted 1,000 (v:v) times with fresh TSB broth and statically cultured at 37°C for 7 h. The bacterial suspension was spotted onto a copper grid and air-dried. Then, the samples were stained using 3% phosphotungstic acid for 2 min and observed using the TEM.

### Bacterial Motility Assay

The swimming assay was performed according to a previous study (Singh et al., 2016) with some modifications. Swimming plates contained 1% tryptone, 0.5% NaCl, and 0.25% agar. For conducting the swimming assays, 1 μL overnight cultures were spotted on the agar plate and incubated at 37°C statically for 12 h. After that, the plate was imaged by using a camera (Nikon D750, Japan).

### Bioinformatic Analysis

Query amino acid sequences of MotA (Houry et al., 2010) (*B. cereus* ATCC14579) and MotB (Cairns et al., 2013) (*B. subtilis* NCIB3610) by BLASTP (ncbi-blast-2.12.0+ -win64). Alignment of amino acid sequences (Supplementary Figure 1) was performed by CLUSTALW^1^ and ESPript 3.0^2^ (Robert and Gouet, 2014).

### Total RNA Isolation, cDNA Synthesis and Reverse Transcription-qPCR Analysis

RNA isolation and purification were performed using the RNeasy Mini Kit (74104, Qiagen, Hilden, Germany) according to the manufacturer’s instructions. RNA concentration and purification were measured with a NanoDrop One Spectrophotometer (Thermo Fisher Scientific, Waltham, MA, United States). For RT-qPCR (reverse transcription-qPCR), purified RNA was used to synthesize cDNA according to the instructions of the PrimeScript™ RT reagent Kit with gDNA Eraser (RR047A, Takara, Shiga, Japan). Primers listed in Supplementary Table 2 were designed by SnapGene^®^ 2.3.2. *udp* (encoding a UDP-*N*-acetylglucosamine 2-epimerase) was used as a reference gene (Reiter et al., 2011). TB Green^®^ *Premix Ex Taq*™ II (Tli RNaseH Plus) (RR820A, Takara, Shiga, Japan) was used for all qPCR reactions. qPCR reactions were performed on a Roche LightCycler^®^ 96 in eight tubes (PCR-0108-LP-RT-C, Axygen, Glendale, AZ, United States) using three-step PCR amplification reaction as follows: 30 s preincubation at 95°C by 1 cycle followed by 45 cycles of denaturation at 95°C for 5 s, annealing at 58°C for 30 s and elongation at 72°C for 30 s, for melting at 95°C for 10 s, 65°C for 60 s, 97°C for 1 s by 1 cycle. The specificity of the reactions was affirmed by melting peaks analysis of the amplified products. Relative expression of *flgE* was calculated by the 2^–ΔΔ*CT*^ (Livak) method (Livak and Schmittgen, 2001) using the difference in Cq (quantification cycle) values of the sample and a calibrator for the target gene and *udp*. Triplicates RT-qPCR reactions were performed for each sample with negative control for three biological repetitions.

### Quantification of Cereulide via Liquid Chromatography Tandem Mass Spectrometry

Cereulide was extracted as described previously with some modifications (Tian et al., 2019). In brief, overnight cultures of *B. cereus* 892-1 were inoculated into 50 mL of LB medium (1:1,000; v:v) and cells were grown at 30°C, 200 rpm for 24 h. Bacteria were collected by centrifugation at 4°C, 8,000 *g* for 5 min, and resuspended in 5 mL methanol (HPLC grade, Guangdong Huankai Co., Ltd., Guangzhou, China). The suspension was cultivated in a shaker at 28°C, 200 rpm overnight. Then, the supernatant was filtered through a 0.22 μm filter, filled with methanol into equal volume, and diluted into a suitable concentration for LC-MS analysis. A Q Exactive Plus Orbitrap LC-MS/MS System (Thermo Fisher Scientific., Waltham, CA, United States) was equipped with an H-ESI (electrospray ionization) II probe source and positive mode was chosen to determine cereulide concentration according to a previous method (In’t Veld et al., 2019). Mass spectrometric characterization of cereulide was performed using a C18 column (ACQUITY UPLC^®^ Peptide BEH, 300A, 1.7 μm, 2.1 mm × 100 mm, 1/pkg). Mass spectrometric detection the ammonium adducts of cereulide at m/z 1,170.7 ([M + NH_4_]^+^) and potassium adducts of valinomycin at m/z 1,128.6 ([M + K]^+^) (Supplementary Figure 4; Seyi-Amole et al., 2020). Methanol and ultrapure water containing 10 mM ammonium formate, both of which contained 0.1% formic acid, were used as eluents A and B. The gradient elution conditions were exhibited in Supplementary Table 3. An injection volume of 5 μL and a flow rate of 10 μL/min were used. MS runtime was 13 min and the retention time (RT) for cereulide and valinomycin was 5.00 and 5.10, respectively. The spray voltage was 3.50 kV. The flow rate for sheath gas was 45 and 10 for aux gas. The temperature for capillary and aux gas heater was 300 and 350°C, respectively. The concentration of cereulide in analytes is calculated by calibration curve, which was obtained by plotting the area ratios of cereulide to valinomycin (internal standard) for different dilutions. Linear regression was applied to give the equation y = 0.00783839x + 0.0686906 with R^2^ = 0.9952; y is the area ratios of cereulide to valinomycin; x is the concentration of cereulide; R^2^ determined the coefficient of the linear regression. The data was acquired and processed by Thermo Xcalibur (v3.5).

## Results

### Identification of Biofilm-Defected Mutants of Emetic *Bacillus cereus* 892-1

**FIGURE 2:**
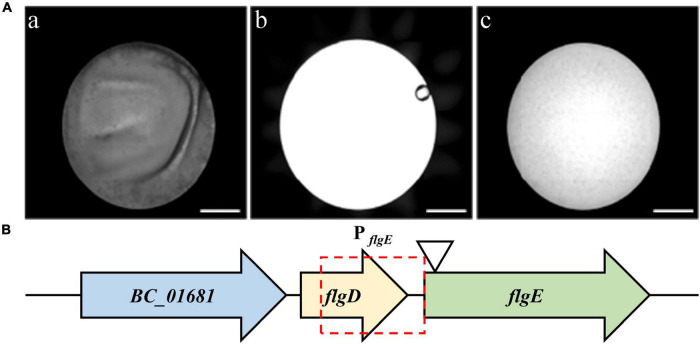
Screening of mutants with biofilm formation defects. **(A)** microplate reader images of wild-type strain (a), only TSB medium (b), and the mutant 3-86 (c). Scale bar = 2 millimeters. **(B)** Diagram showing the transposon insertion site of the defective mutant. Potential promoter of *flgE* is indicated by the red frame. The position of the TnYLB-1 transposon in *flgE* on the chromosome of *B. cereus* 892-1 is indicated by the triangle.

In total, 500 chloromycetin-resistant and erythromycin-sensitive mutants were identified, which were then screened for the identification of potential mutants with defects in biofilm formation. Among them, one mutant, designated 3-86, presented an obvious biofilm formation defect (Figure 2A). After reverse PCR, sequencing, and local blast, the transposon was proved to insert into the gene *flgE* (Figure 2B).

### *flgE* Positively Regulates Biofilm Formation in Emetic *Bacillus cereus* 892-1

**FIGURE 3:**
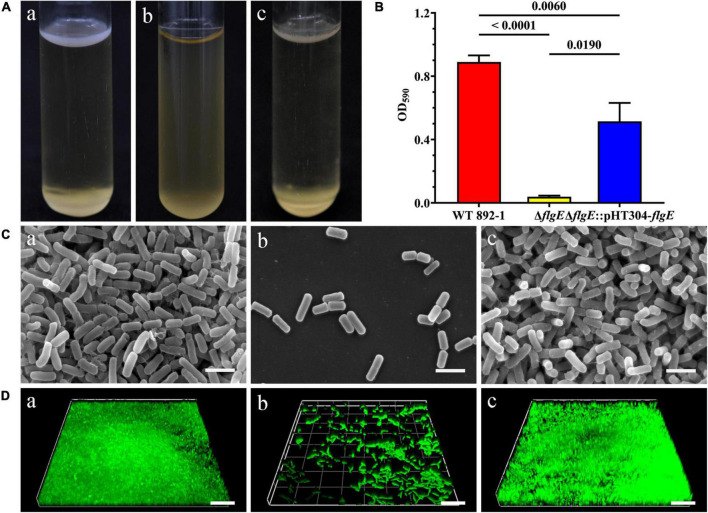
*flgE* is essential for biofilm formation in emetic *B. cereus* 892-1. **(A)** Pellicle formation by wild-type strain (a), Δ*flgE* (b), and Δ*flgE*:pHT304-*flgE* (c). **(B)** Ring formation by wild-type strain 892-1, Δ*flgE*, and Δ*flgE*:pHT304-*flgE*. *P* values were calculated using the Student’s *t*-test. Error bars represent the standard deviation of the mean. **(C)** SEM images of biofilms formed by (a) wild-type, (b) Δ*flgE*, and (c) Δ*flgE*:pHT304-*flgE*. The scale bars represent three microns. **(D)** CLSM images of biofilms formed by wild-type (a), Δ*flgE* (b), and Δ*flgE*:pHT304-*flgE* (c). The scale bars represent 30 microns.

To further illustrate the role of *flgE* in biofilm formation, we compared the pellicle formation of wild-type strain with Δ*flgE* and complementary strain. Wild-type cells can form a pellicle in the air-liquid interface, while Δ*flgE* strain cannot (Figure 3A). As expected, the complementary strain restored a comparable level in its ability to form a pellicle. Furthermore, the amount of the ring was significantly reduced in Δ*flgE*; however, the ring of the complementary strain (Δ*flgE*:pHT304-*flgE*) was largely restored, although it did not reach the wild-type level (Figure 3B). Through observation by an SEM, the wild-type and complementary strains showed a dense biofilm community (Figure 3C). In contrast, only sparse cells of the mutant strain remained on the grid. Besides, biofilms were imaged by CLSM. The results are the same as SEM. The wild-type and complementary strains had dense biofilm structure, while Δ*flgE* only had scattered bacteria (Figure 3D).

### *flgE* Is Necessary for Flagella Synthesis and Swimming Ability

**FIGURE 4:**
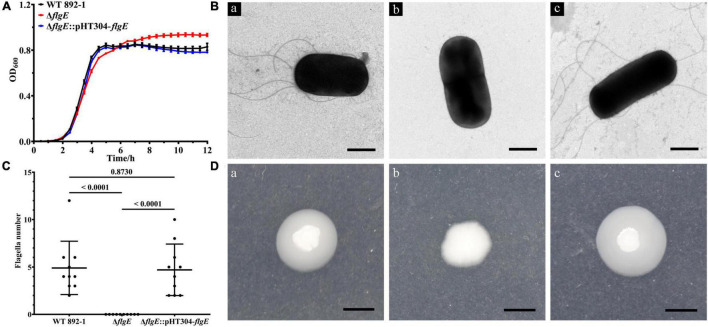
*flgE* is important for flagella synthesis and function. **(A)** Growth curves of wild-type, Δ*flgE*, and Δ*flgE*:pHT304-*flgE*. **(B)** TEM images of flagella produced in the wild-type (a), Δ*flgE* (b), and Δ*flgE*:pHT304-*flgE* (c). Scale bars represent one micron. **(C)** The number of flagella in wild-type strain, Δ*flgE*, and Δ*flgE*:pHT304-*flgE*. The number of flagella was calculated by 10 bacteria. **(D)** Swimming motility of wild-type (a), Δ*flgE* (b), and Δ*flgE*:pHT304-*flgE* (c). Scale bars represent zero point five centimeter.

To exclude the possibility that the biofilm formation defect is due to the differential growth rate, we monitored the growth of different bacteria for 12 h and found that the growth rate of Δ*flgE* had no obvious difference compared with wild-type and Δ*flgE*:pHT304-*flgE* at the early stage and was a little bit faster than other strains at the stationary stage (Figure 4A). To verify the role of *flgE* in flagella synthesis or assembly in *B. cereus* 892-1, the cells of wild-type, Δ*flgE*, and Δ*flgE*:pHT304-*flgE* were inspected by a TEM. No flagella could be found in Δ*flgE*. In contrast, either the wild-type or Δ*flgE*:pHT304-*flgE* had obvious flagella (Figure 4B), and there was no difference in quantity between the wild-type and Δ*flgE*:pHT304-*flgE* (Figure 4C). Due to the loss of flagella, Δ*flgE* could not swim as no outward movement can be observed (Figure 4D).

### Swimming Ability Is Not Necessary for Biofilm Formation in Emetic *Bacillus cereus* 892-1

**FIGURE 5:**
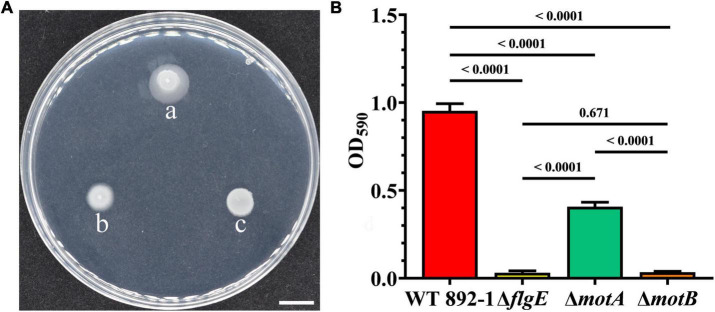
Swimming ability is not necessary for biofilm formation. **(A)** Swimming motility of wild-type (a), Δ*motB* (b), and Δ*motA* (c). Scale bars represent one centimeter. **(B)** Ring formation by wild-type strain, Δ*flgE*, Δ*motA*, and Δ*motB*. *P* values were calculated using the Student’s *t*-test, and used to determine statistical significance between each mutant and the wild-type strain. Error bars represent the standard deviation of the mean.

To test the swimming ability or flagella itself is important for biofilm formation in emetic *B. cereus*, two flagellar paralytic strains were constructed and the biofilm formation ability was measured. As expected, Δ*motA* and Δ*motB* lost swimming ability in motility assay (Figure 5A). Surprisingly, pellicle could also be formed in Δ*motA* instead of in Δ*motB* (Supplementary Figure 2). The amount of ring in Δ*motA* was also significantly higher than that in Δ*flgE* or Δ*motB*, but lower than that in the wild-type strain (Figure 5B), demonstrating that swimming ability contributes to biofilm formation but is not necessary for biofilm formation. Δ*motB* completely lost the ability to form a biofilm, and has no significant difference in biofilm formation compared with Δ*flgE*, indicating that flagellar structure itself does not play a scaffold-role in emetic *B. cereus* 892-1.

### Loss of *flgE* Significantly Decreased Cereulide Production in *Bacillus cereus* 892-1

**FIGURE 6:**
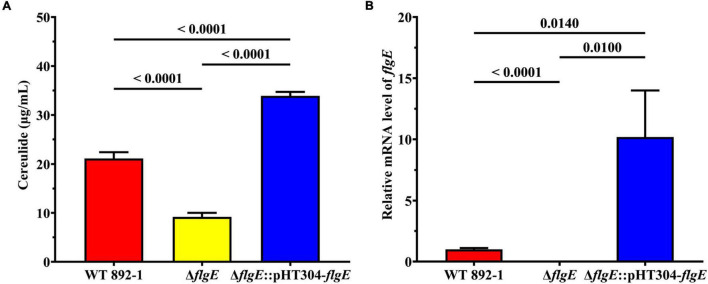
Deletion of *flgE* significantly decreased cereulide production. **(A)** Cereulide production by wild-type strain, Δ*flgE* and Δ*flgE*:pHT304-*flgE*. **(B)** Relative mRNA levels of *flgE* in wild-type strain, Δ*flgE*, and Δ*flgE*:pHT304-*flgE*. *P* values were calculated using the Student’s *t*-test. Error bars represent the standard deviation of the mean.

Since 892-1 is an emetic strain, we also evaluated cereulide production in different strains by LC-MS/MS. The concentration range of cereulide was 19.327–22.757, 8.1586–10.312, and 32.417 to 35.294 μg/mL in WT 892-1, Δ*flgE* and Δ*flgE*:pHT304*-flgE*, respectively (Figure 6A; Supplementary Table 4). In conclusion, cereulide production was significantly decreased in Δ*flgE*, with a reduction of approximately 60% compared with the wild-type strain. In addition, Δ*flgE*:pHT304*-flgE* produced more cereulide; therefore, the transcriptional levels of *flgE* were monitored in the bacterial logarithmic phase. The relative expression level of *flgE* was obviously increased in Δ*flgE*:pHT304-*flgE* compared with wild-type (Figure 6B).

## Discussion

*Bacillus cereus* can contaminate different type of foods (Messelhäusser et al., 2010; Kim et al., 2016; Esteban-Cuesta et al., 2018; Fasolato et al., 2018; Park et al., 2018; Yu et al., 2019, 2020). Notably, a relatively high prevalence of emetic strains exists in dairy products, including cow milk, and pasteurized and ultrahigh-temperature treated milk products (Wijnands et al., 2006; Messelhäusser et al., 2014; Chaves et al., 2017; Owusu-Kwarteng et al., 2017; Gao et al., 2018; Walser et al., 2021). Emetic strains can produce highly heat resistant and acid-stable cereulide which brings a great threat to human health (Rouzeau-Szynalski et al., 2020). It is worth noting that *B. cereus* contamination occurred in dairy production was largely due to the biofilm formation (Teh et al., 2012; Yobouet et al., 2014; Reda, 2019; Wang et al., 2019), indicating a possible transmission of emetic *B. cereus* from food processing environment to human infection (Kuroki et al., 2009). Besides, cereulide showed a heightened affinity to lipid components of milk samples (Walser et al., 2021). Therefore, it is important to analyze the mechanism of emetic *B. cereus* biofilm formation which can be used to inhibit the formation of emetic *B. cereus* biofilms in the food industry, especially in dairy production.

In this study, we showed that *flgE* identified by the transposon mutagenesis is essential for the biofilm formation of emetic *B. cereus* 892-1. In the stationary phase, the growth rate of *flgE* knockout strain was higher than that of the wild-type strain and complementary strain. It was speculated that flagella synthesis needed energy, so bacterial growth was promoted in Δ*flgE* which cannot form flagella (Hölscher et al., 2015). Bacterial flagella are closely related to biofilm formation (Guttenplan and Kearns, 2013). In *E. coli*, half of the mutants with defects in biofilm formation are defective in flagellar function (Pratt and Kolter, 1998). Plenty of studies demonstrated that the destruction of flagella affects the phenotype of biofilm by affecting flagella-mediated motility in different species (O’Toole and Kolter, 1998; Pratt and Kolter, 1998; Lee et al., 2004; Hossain and Tsuyumu, 2006). Besides, it is proved that the signal transmitted by flagella can stimulate biofilm formation (Belas, 2014). In *B. subtilis*, inhibiting flagellar movement by destroying flagellar stator protein MotB, over-expressing site-directed EpsE mutant, or using flagellin antibody can stimulate the generation of biofilm matrix, and the appearance of colony biofilm phenotype by activating DegS-DegU two-component system (Cairns et al., 2013). In *V. cholerae*, the deletion of flagellar filament structure can stimulate the biofilm formation by increasing the level of second-messenger cyclic diguanylate (c-di-GMP), which requires the participation of flagellar stator protein (Wu et al., 2020). In *P. aeruginosa*, c-di-GMP plays an important role in the regulation of flagella and biofilm. The flagellar stator protein MotCD has been proved to interact with diguanylate cyclase SadC to activate the activity of SadC, thus stimulating the production of c-di-GMP, inhibiting the swarm movement, and promoting the formation of biofilm (Caiazza et al., 2007; Baker et al., 2019). In contrast, it has also been suggested that flagella-mediated motility is not necessary for biofilm formation. In *B. subtilis*, the immobile cells caused by destroying flagellin protein Hag can reach the gas-liquid interface by Brownian movement and then form a biofilm (Hölscher et al., 2015). To investigate the participation of flagella-mediated motility in the biofilm formation of emetic strain 892-1, we mutated the flagellar stator proteins which are reported to control swimming ability without affecting flagellar structure (Houry et al., 2010; Cairns et al., 2013). To our surprise, the biofilm of Δ*motA* significantly decreased when compared with the wild-type cell (Figure 5B and Supplementary Figure 2). In contrast, Δ*motB* completely lost the ability to form the biofilm. Together, these results indicated that *flgE*, apart from its flagella-mediated swimming ability, plays other unknown regulatory roles that contribute to biofilm formation in 892-1. Although the flagellar structure is considered to be able to maintain the stability of biofilm structure in many other species such as in *P. aeruginosa* (Ozer et al., 2021), *Helicobacter pylori* (Hathroubi et al., 2018), and *Geobacter sulfurreducens* (Liu et al., 2019), Δ*motB* had no obvious difference in biofilm formation compared with Δ*flgE* (Figure 5B and Supplementary Figure 2), indicating that flagella themselves do not play a scaffold-role in 892-1.

Moreover, the deletion of *flgE* not only reduced biofilm formation, but also significantly down-regulated cereulide production (Figure 6A). To our surprise, the amount of cereulide in complementary strain was higher than the wild-type strain. We monitored the transcriptional levels of *flgE*, the expression level of *flgE* in the complementary strain was significantly higher than the wild-type strain (Figure 6B), indicating the difference in the production of cereulide between the wild-type strain and supplementary strain may be caused by the differential expression of *flgE*. Therefore, we speculated that *flgE* may serve as an important contributor to both biofilm formation and cereulide production, which suggests that the two phenotypes are possibly governed by a common system within the cell. The potential regulatory effect of flagella in mediating virulence or pathogenicity has been reported widely (Haiko and Westerlund-Wikström, 2013; Stevenson et al., 2015). In *B. cereus*, *flhF*, which controls the arrangement of flagella, is important in cell migration, especially swarming motility (Salvetti et al., 2007). Deletion of *flhF* significantly affects the pathogenicity of *B. cereus*, resulting in a reduction of infection *in vivo* (Mazzantini et al., 2016). Quorum sensing (QS) is an important system in cell-cell communication that is involved in many biological processes, including biofilm formation and virulence (Rutherford and Bassler, 2012). In *V. cholerae*, QS autoinducers cholerae autoinducer-1 (CAI-1) and autoinducer-2 (AI-2) cannot bind to the kinases CqsS and LuxPQ on the cell membrane at a low cell density, resulting in the activation of biofilm formation and virulence (Bridges and Bassler, 2019). The relationship between biofilm formation and cereulide production in the emetic strain of *B. cereus* is still unclear and the mechanism of *flgE* in these two processes needs to be further investigated in the future.

## Conclusion

In this study, we showed that flagellar hook protein FlgE is critical in biofilm formation in emetic *B. cereus*. The potential role of FlgE does not depend on the scaffold-role of flagella in biofilm formation. Instead, swimming ability contributes to biofilm formation, but is not necessary for it. Moreover, loss of *flgE* also reduced cereulide production, demonstrating the dual role of the flagellar hook protein in emetic *B. cereus*. Therefore, FlgE can be used as a target for the control of food contamination and poisoning incidents caused by emetic *B. cereus*.

## Data Availability Statement

The original contributions presented in the study are included in the article/Supplementary Material, further inquiries can be directed to the corresponding authors.

## Author Contributions

YD, QW, JW, JZ, MC, YL, and NC conceived the project and designed the experiments. YL, NC, XY, SY, and XL performed the experiments. YD and JW supervised the project. YL, NC, JW, and YD analyzed the data and wrote the manuscript. QW, JW, ZZ, YZ, and YD complemented the writing. All authors contributed to the article and approved the submitted version.

## Conflict of Interest

The authors declare that the research was conducted in the absence of any commercial or financial relationships that could be construed as a potential conflict of interest.

## Publisher’s Note

All claims expressed in this article are solely those of the authors and do not necessarily represent those of their affiliated organizations, or those of the publisher, the editors and the reviewers. Any product that may be evaluated in this article, or claim that may be made by its manufacturer, is not guaranteed or endorsed by the publisher.

## Footnotes

1
https://www.genome.jp/tools-bin/clustalw

2
https://espript.ibcp.fr/ESPript/cgi-bin/ESPript.cgi
