# Supplementary material for: A Flagella Hook Coding Gene flgE Positively Affects Biofilm Formation and Cereulide Production in Emetic Bacillus cereus
Source: Front Microbiol. 2022 Jun 10;13:897836. doi: 10.3389/fmicb.2022.897836 (PMC9226606; doi:10.3389/fmicb.2022.897836)
Supplement: Supplementary file 1 [file Data_Sheet_1.docx]

Supplementary Materials

# Supplementary Figures and Tables


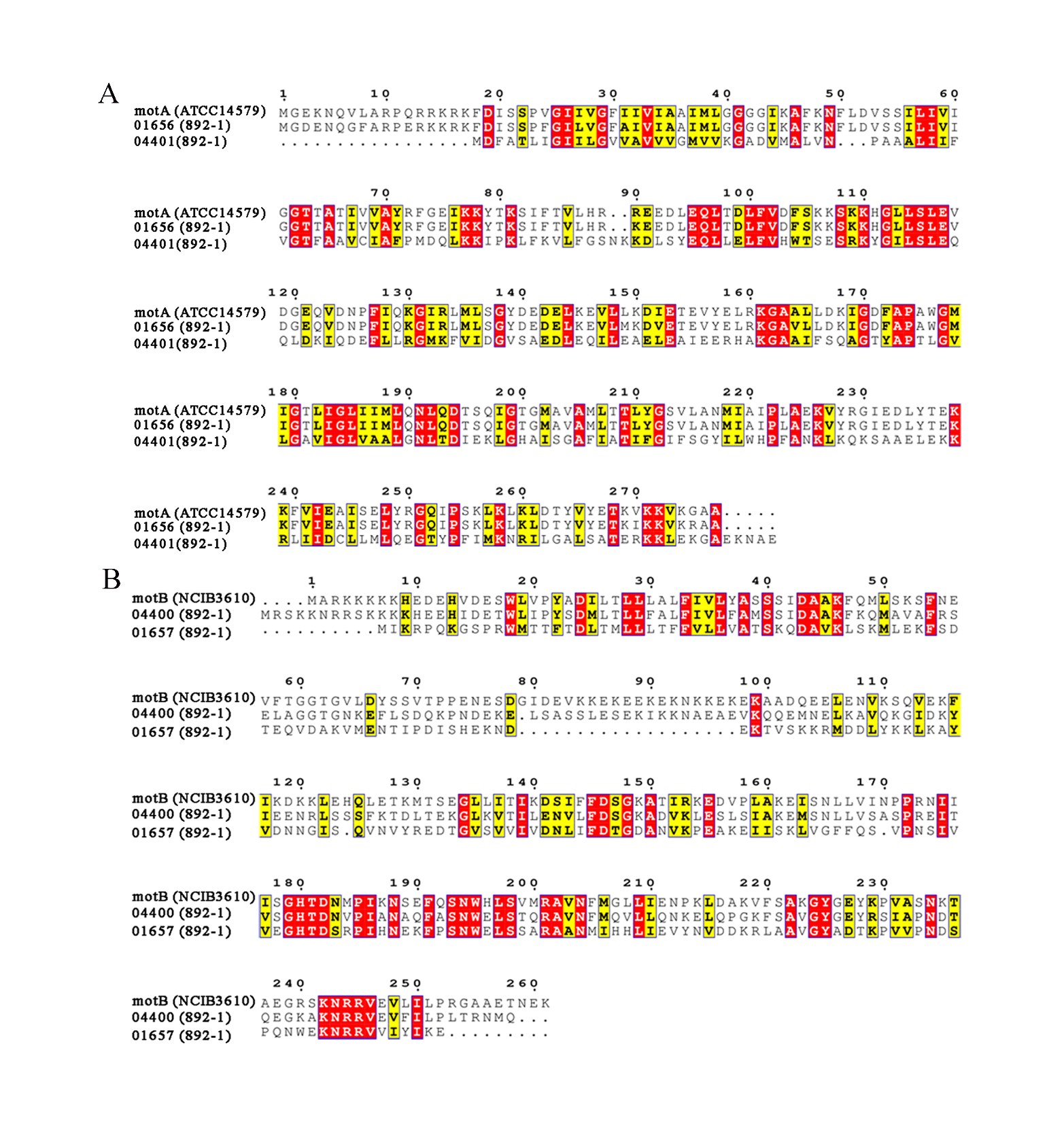


**Supplementary Figure 1.** Alignment of the amino acid sequences of MotA and MotB in *B. cereus* 892-1. (A) Alignment of amino acid sequences of MotA of *B. cereus* ATCC14579 with *B. cereus* 892-1 (Locus tag 01656 and 04401). (B) Alignment of amino acid sequences of MotB of *B. subtilis* NCIB3610 with *B. cereus* 892-1 (Locus tag 04400 and 01657). Alignment of amino acid sequences were used by CLUSTALW and ESPript (Robert and Gouet, 2014). Red color indicates conserved sequence of amino acids appearing in all aligned sequences, and yellow color indicates conserved amino acids appearing in two sequences. Gene *01656* and *04400* were selected for knockout.


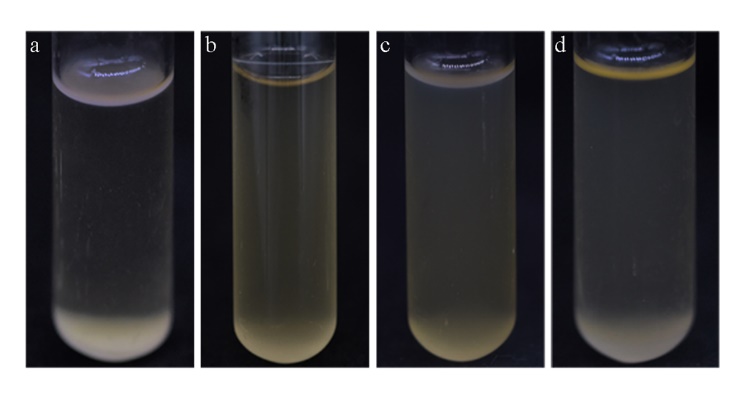


**Supplementary Figure 2.** Swimming ability is not necessary for biofilm formation. Pellicle formation by wild-type strain (a), Δ*flgE* (b), Δ*motA* (c), and Δ*motB* (d).


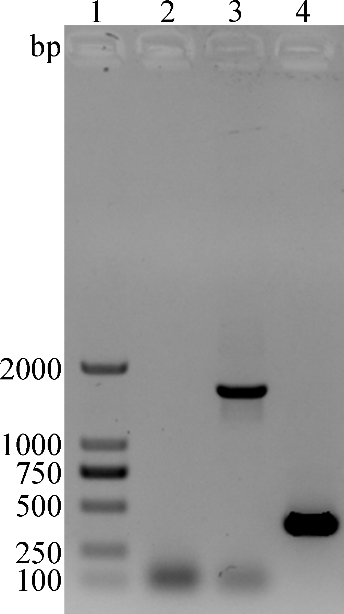


**Supplementary Figure 3.** Agarose gel electrophoresis of PCR products of wild type strain and Δ*flgE.* Lane 1: 5000 base pair DNA marker; lane 2: PCR products without DNA template; lane 3: wild type strain; lane 4: Δ*flgE*. The PCR products of wild type strain and Δ*flgE* are 1638 bp and 414 bp, respectively.


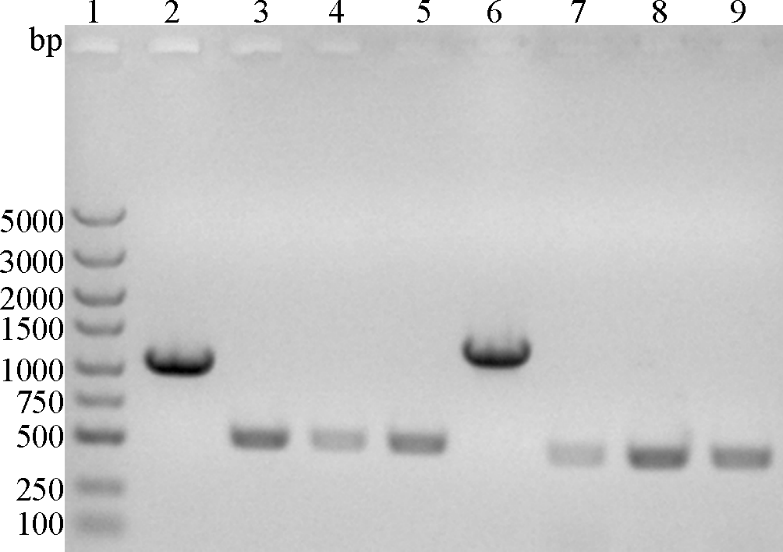


# Supplementary Figure 4. Agarose gel electrophoresis of PCR products of wild type strain, Δ*motA*, and Δ*motB.* Lane1: 5000 base pair DNA marker; lane 2 and lane 6: wild type strain; lane 3, 4, and 5: *motA* mutants; lane 7, 8, and 9: *motB* mutants. The PCR products of *motA* and *motB* mutants are 525 bp and 474 bp, respectively. The PCR products of wild type strain are 1086 bp and 1249 bp in lane 2 and lane 6, respectively.


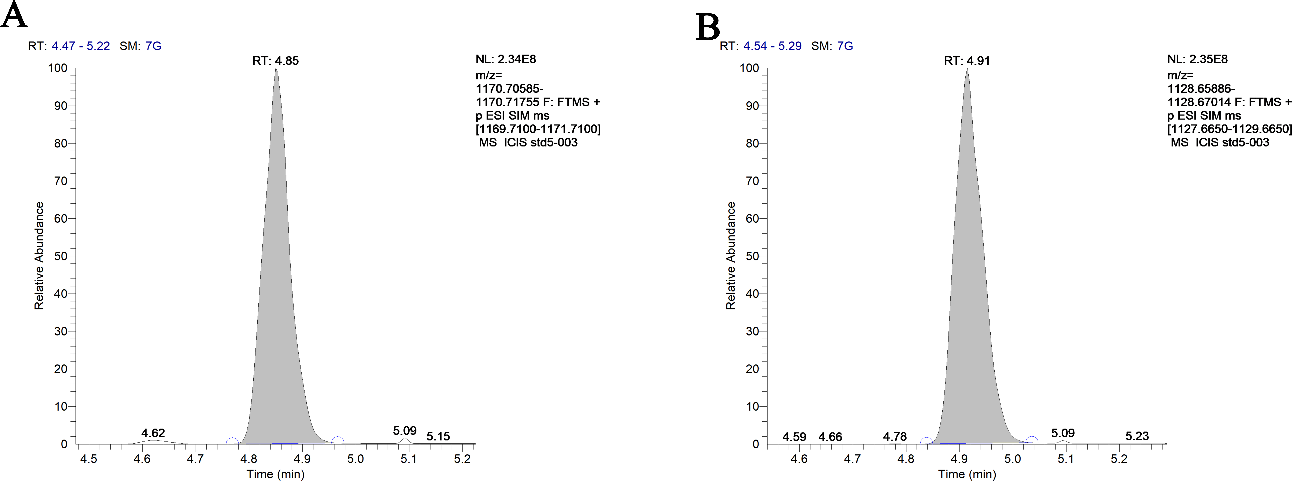


**Supplementary Figure 5.** LC-MS/MS chromatograms of cereulide (A) and valinomycin (B).

**Supplementary Table 1** Strains and plasmids used in this study

| **Strain/plasmid** | **Description** | **From** |
| --- | --- | --- |
| Strains |  |  |
| *Bacillus cereus* 892-1 | Isolated from pasteurized milk | (Gao et al., 2018) |
| Δ*flgE* | Knocked out the gene *flgE* | This study |
| Δ*flgE*::pHT304-*flgE* | Ectopic expression gene *flgE* in Δ*flgE* | This study |
| Δ*motA* | Knocked out the gene *motA* | This study |
| Δ*motB* | Knocked out the gene *motB* | This study |
| Plasmids |  |  |
| pMarA | Construct transposon mutagenesis library | (Gao et al., 2017) |
| pMarA-cat | Construct transposon mutagenesis library | This study |
| pHT304 | Complement gene | (Arantes and Lereclus, 1991) |
| pHt304-TS | Knockout gene | (Zhu et al., 2011) |
| pHT304-TS-*flgE* | Knockout the gene *flgE* | This study |
| pHT304-TS-*motA* | Knockout the gene *motA* | This study |
| pHT304-TS-*motB* | Knockout the gene *motB* | This study |
| pHT304-*flgE* | Complement the gene *flgE* | This study |

**Supplementary Table 2** Primers used in this study

| **Primer** | **Sequence (5’-3’)** | **Use** |
| --- | --- | --- |
| Reverse-PCR | F: TACCTAGATTTAGATGTCTAAAAAGCTTTAACTACAAGCTTTTTAGACATCTA  R: TTATTATTTCCTTCCTCTTTTCTACAGTATTTAAAGATACCCCAAGAAGCTAA | Amplify linearized vector |
| Cat-In | F: TAGAAAAGAGGAAGGAAATAATAAATGGAGAAAAAAATCACTGG  R: TTTTAGACATCTAAATCTAGGTATTACGCCCCGCCCTGCCACTC | Amplify chloramphenicol gene |
| oIPCR | 1: GCTTGTAAATTCTATCATAATTG  2: AGGGAATCATTTGAAGGTTGG | Confirm transposon insertion site (Le Breton et al., 2006) |
| Himar | F: GTCGACGCAGATTCCGGTCTAACAAAG  R: TGGAGCAATTCGGACGATTG | Amplify transposase gene |
| Cat | F: GCCACTCATCGCAGTACTGTTGTAA  R: ACCGTTGATATATCCCAATGGCATC | Amplify partial chloramphenicol gene |
| Erm | F: CTTTGAAATCGGCTCAGGAAAAGGC  R: ATCGTCAATTCCTGCATGTTTTAAG | Amplify partial erythromycin gene |
| *flgE*-up | F: AAGCTTGCATGCCTGCAGGTCGACGAAGAAACGCGTTGAAATCATTTTTGATGAAT  R: CTTGATTATATAAATGTATGGACACTCCTTATGAATTTGTAGTTTTTTC | Knockout gene *flgE* |
| *flgE*-down | F: CCATACATTTATATAATCAAGAAAAATTGTAATGTAGTAGAAAGGGTTTTTTATT  R: GTAAAACGACGGCCAGTGAATTCTACAGTCCGCTGCTCAACCTCACT | Knockout gene *flgE* |
| *flgE*-YZ | F: GGAATGAAGTTTTTAGGGAAGTACG  R: CTGGTAAGTATACATGCTCTTCTCG | Identify gene *flgE* knockout |
| *flgE-*HB | F: GCTTGCATGCCTGCAGGTCGACGTTTCCAGCACCAAGATCC  R: TAAAACGACGGCCAGTGAATTCTTATCGAATTAAGTTTACAAC | Complement gene *flgE* |
| *motA*-up | F: AAGCTTGCATGCCTGCAGGTCGACTGAATTTTTACCTCAAGCTTATCAAGTGCGTTAG  R: AGTTTTGAAGATTCCTGCCCTTTCCTTTTCATTTTTATAAAACATTTTTTTA | Knockout gene *motA* |
| *motA*-down | F: GGGCAGGAATCTTCAAAACTTGCAAGACACATCGCAAATTGGTACAGGGA  R: GTTGTAAAACGACGGCCAGTGAATTCTATACATTCACTTGACTAATACCGTTATTATC | Knockout gene *motA* |
| *motA*-YZ | F: AAGGTTCAACTAAAATGGCCATTAG  R: TTAATTTAGACGGAATTTGTCCACG | Identify gene *motA* knockout |
| *motB*-up | F: AAGCTTGCATGCCTGCAGGTCGACCGTACTTGATTTAGATGAAACTGTTTTAGATAAC  R: TGCGGAGTAAAAGAGGAGGGCACATGCCATCCTCTTTTATTTTGCATCAAATG | Knockout gene *motB* |
| *motB*-down | F: CCCTCCTCTTTTACTCCGCATTTTTTTCTGCCCCTTTTTCAAGCTTTTTG  R: TGTAAAACGACGGCCAGTGAATTCCTTTTTGGTTCTAATAAAAAAGATTTAAGTTATG | Knockout gene *motB* |
| *motB*-YZ | F: AGTCTGTAAAGGACCGAAACCAAGC  R: AGTTAGGTCACGCTATTTCGGGTGC | Identify gene *motB* knockout |
| 304TS | F: CTTCCGGCTCGTATGTTGTG  R: TGTGCTGCAAGGCGATTAAG | Identification of recombinant plasmid |
| *flgE*-RT | F: CGGCAATGGAAGGTAATGGTTTC  R: ACATTACCAGTTGATGAATCCGC | RT-PCR assay for *flgE* |
| *udp*-RT | F: CGGAGCTTCCTCTTGTACAC  R: CCTGTTGTTCGTGAAACTGC | RT-PCR assay for *udp* |

# Supplementary Table 3 Gradient elution conditions used in this study for cereulide quantification

| **Number** | **Time** | **Flow rate (ml/min)** | **A (%)** | **B (%)** |
| --- | --- | --- | --- | --- |
| 1 | 0.0 | run | | |
| 2 | 0.0 | 0.3 | 30.0 | 70.0 |
| 3 | 2.0 | 0.3 | 10.0 | 90.0 |
| 4 | 5.0 | 0.3 | 5.0 | 95.0 |
| 5 | 7.0 | 0.3 | 5.0 | 95.0 |
| 6 | 8.0 | 0.3 | 30.0 | 70.0 |
| 7 | 13.0 | stop run | | |

A: 0.1% [formic](javascript:;) [acid](javascript:;) in methanol (LC/MS grade).

B: 10 mM [ammonium](javascript:;) [formate](javascript:;) and 0.1% [formic](javascript:;) [acid](javascript:;) in ultrapure water.

# Supplementary Table 4 Liquid chromatography tandem mass spectrometry analysis of cereulide in WT/Δ*flgE*/Δ*flgE*::pHT304-*flgE*

| **Sample name^a^** | **Area^b^** | **ISTD area^c^** | **Calculated amount^d^**  **(ng/ml)** | **RT^e^** | **Concentration^f^**  **(μg/ml)** |
| --- | --- | --- | --- | --- | --- |
| WT-1-1 | 223371788 | 904095797 | 22.757 | 4.84 | 22.757 |
| WT-1-2 | 223208092 | 926708455 | 21.965 | 4.85 | 21.965 |
| WT-1-3 | 206372849 | 841307886 | 22.531 | 4.85 | 22.531 |
| WT-2-1 | 201695007 | 851529332 | 21.455 | 4.86 | 21.455 |
| WT-2-2 | 207226406 | 863142102 | 21.866 | 4.86 | 21.866 |
| WT-2-3 | 196606608 | 874721247 | 19.912 | 4.87 | 19.912 |
| WT-3-1 | 198972516 | 850094484 | 21.097 | 4.86 | 21.097 |
| WT-3-2 | 195943688 | 889905470 | 19.327 | 4.86 | 19.327 |
| WT-3-3 | 196039969 | 887549060 | 19.416 | 4.87 | 19.416 |
| Δ*flgE*-1-1 | 617344667 | 845683451 | 84.367 | 4.86 | 8.4367 |
| Δ*flgE*-1-2 | 621953598 | 867080601 | 82.747 | 4.84 | 8.2747 |
| Δ*flgE*-1-3 | 613407224 | 866154284 | 81.586 | 4.84 | 8.1586 |
| Δ*flgE*-2-1 | 736402718 | 887478388 | 97.096 | 4.84 | 9.7096 |
| Δ*flgE*-2-2 | 753076670 | 858712134 | 103.120 | 4.84 | 10.312 |
| Δ*flgE*-2-3 | 764925250 | 917952833 | 97.546 | 4.84 | 9.7546 |
| Δ*flgE*-3-1 | 718595188 | 857719462 | 98.120 | 4.84 | 9.812 |
| Δ*flgE*-3-2 | 720294519 | 910673501 | 92.143 | 4.84 | 9.2143 |
| Δ*flgE*-3-3 | 707384077 | 916314756 | 89.725 | 4.84 | 8.9725 |
| Δ*flgE*::pHT304-*flgE*-1-1 | 330441743 | 947373883 | 35.735 | 5.0 | 35.735 |
| Δ*flgE*::pHT304-*flgE*-1-2 | 317136634 | 958935460 | 33.429 | 5.00 | 33.429 |
| Δ*flgE*::pHT304-*flgE*-1-3 | 314243637 | 909953714 | 35.294 | 5.00 | 35.294 |
| Δ*flgE*::pHT304-*flgE*-2-1 | 309085378 | 923671084 | 33.927 | 5.00 | 33.927 |
| Δ*flgE*::pHT304-*flgE*-2-2 | 314980685 | 951642199 | 33.463 | 5.00 | 33.463 |
| Δ*flgE*::pHT304-*flgE*-2-3 | 305246942 | 920513779 | 33.542 | 5.01 | 33.542 |
| Δ*flgE*::pHT304-*flgE*-3-1 | 295562045 | 879329141 | 34.118 | 5.00 | 34.118 |
| Δ*flgE*::pHT304-*flgE*-3-2 | 281532029 | 872191247 | 32.417 | 5.01 | 32.417 |
| Δ*flgE*::pHT304-*flgE*-3-3 | 279865460 | 847710311 | 33.355 | 5.01 | 33.355 |

^a^ Three technical and biological repeats of wild-type (WT), Δ*flgE*, and Δ*flgE*::pHT304-*flgE*.

^b^ [Response](javascript:;) [value](javascript:;) of cereulide.

^c^ [Response](javascript:;) [value](javascript:;) of [valinomycin](javascript:;); ISTD = Internal Standard.

^d^ Amount calculated by the equation y = 0.00783839x + 0.0686906 with R^2^ = 0.9952 (y = Area/ISTD area, x = amount).

^e^ Retention time.

^f^ Cereulide concentration of samples calculated by dilution ratio (1:1000 for WT and Δ*flgE*::pHT304-*flgE*, and 1:100 for Δ*flgE*).

# References

Arantes, O., and Lereclus, D. (1991). Construction of cloning vectors for *Bacillus thuringiensis*. *Gene* 108(1), 115-119. doi: 10.1016/0378-1119(91)90495-w

Gao, T., Ding, Y., Wu, Q., Wang, J., Zhang, J., Yu, S., et al. (2018). Prevalence, virulence genes, antimicrobial susceptibility, and genetic diversity of *Bacillus cereus* isolated from pasteurized milk in China. *Front Microbiol*. 9, 533. doi: 10.3389/fmicb.2018.00533

Gao, T., Li, Y., Ding, M., Chai, Y., and Wang, Q. (2017). The phosphotransferase system gene *ptsI* in *Bacillus cereus* regulates expression of *sodA2* and contributes to colonization of wheat roots. *Res Microbiol*. 168(6)**,** 524-535. doi: 10.1016/j.resmic.2017.04.003.

Le Breton, Y., Mohapatra, N.P., and Haldenwang, W.G. (2006). In vivo random mutagenesis of *Bacillus subtilis* by use of TnYLB-1, a mariner-based transposon. *Appl Environ Microbiol* 72(1)**,** 327-333. doi: 10.1128/AEM.72.1.327-333.2006.

Robert, X., and Gouet, P. (2014). Deciphering key features in protein structures with the new ENDscript server. *Nucleic Acids Res*. 42(W1)**,** W320-W324. doi: 10.1093/nar/gku316.

Zhu, Y., Ji, F., Shang, H., Zhu, Q., Wang, P., Xu, C., et al. (2011). Gene clusters located on two large plasmids determine spore crystal association (SCA) in *Bacillus thuringiensis* subsp. finitimus strain YBT-020. *PLoS One* 6(11)**,** e27164. doi: 10.1371/journal.pone.0027164.
